# Assessment of knowledge, perception, preventive practices and effects of COVID-19 among Nigerians: a cross-sectional study

**DOI:** 10.11604/pamj.2022.41.102.30262

**Published:** 2022-02-04

**Authors:** Henshaw Uchechi Okoroiwu, Ifeyinwa Maryann Okafor, Chidiebere Peter Echieh, Christopher Ogar Ogar, Dennis Akongfe Abunimye, Ikenna Kingsley Uchendu

**Affiliations:** 1 Hematology Unit, Department of Medical Laboratory Science, University of Calabar, Calabar, Nigeria,; 2 Division of Cardiothoracic Surgery, University of Calabar Teaching Hospital, Calabar, Nigeria,; 3 Department of Medical Laboratory Science, University of Nigeria, Enugu Campus, Enugu, Nigeria

**Keywords:** COVID-19, awareness, knowledge, prevention practice, perception

## Abstract

**Introduction:**

the coronavirus disease 2019 (COVID-19) has become a disease of global public health concern. The current cumulative cases in Nigeria are high. The effective control of the pandemic is dependent on knowledge, attitude and willingness of people to adapt their life to the new reality. The purpose of this study is to determine the knowledge, perception, preventive practice, and effect of COVID-19 on the respondents.

**Methods:**

we conducted a cross sectional, online survey-based study (Google form) from May 25^th^, 2020 to June 25^th^, 2020. The survey questionnaire consisted of demographic characteristics, 13 items on knowledge, 6 items on preventive practices, 13 items on perception. Descriptive statistics, t-test, one-way ANOVA and bivariate logistic regression were carried out.

**Results:**

the correct overall knowledge was 98.03% with average score of 11.7 ± 1.0 (9-13). Knowledge scores were comparable in all demographics except marital status. Most of the participants practiced preventive procedures such as wearing face mask (95.1%), use of alcohol based hand sanitizer (78.9%), avoidance of worship centers (62.4%) and regular hand washing. There were several perceptions ranging from rumors to unfounded scientific claims. Gender, level of education, monthly income and Christian denomination were predictors of use of hand sanitizer while age, level of education, monthly income, Christian denomination and region were predictors of attendance of worship centers during lockdown.

**Conclusion:**

the results of this study suggest government should gain trust of citizens to translate knowledge to practice and full compliance of regulations.

## Introduction

An outbreak of pneumonia of unknown origin was reported in the city of Wuhan in China´s Hubei Province on December 31^st^, 2019 [1,2]. On February 11^th^, 2020 the World Health Organization (WHO) named the disease coronavirus disease 2019 (COVID-19) while the pathogen was named severe acute respiratory syndrome coronavirus 2 (SARS-CoV-2) by the *coronaviridae* study group of the International Committee on Taxonomy of Viruses (ICTV) [3-5]. The pathogen SARS-CoV-2 is an enveloped, positive-sense single stranded virus belonging to the realm; *riboviria*, order; *nidovirales*, suborder: *cornidovirineae*, family: *coronaviridae*, subfamily: *orthocoronavirinae*, genus: *betacoronavirus* and subgenus: *sarbecovirus* [4,6]. The virus spreads through human to human mainly via respiratory droplets produced when an infected person coughs or sneezes and body contacts [7,8]. More so, fomites may be a large source of transmission as the virus has been reported to persist on surfaces for up to 96 hours [9]. The incubation period of 5.2 days (95% CI; 4.1-7.0 days) ranging from 1-14 days has been reported [10] and 97.5% of those who develop symptoms will do so within 11 days [11].

Incubation periods of 19 or 24 days have been reported [8,12], although case definitions typically rely on 14 day window period [13] under the conservative assumption that 101 out of every 10,000 cases (99^th^ percentile) will develop symptoms after 14 days of active monitoring. The main symptoms of the disease include fever (98.6%), fatigue (69.6%), dry cough (59.4%) and dyspnea (43.0%) [2,14]. Viral loads were highest in bronchoalveolar lavage samples (93%) and bronchoalveolar lavage is mainly used to collect samples [15]. Older persons with comorbidities are more likely to be infected with COVID-19 and usually have the worst outcomes. Severe cases may result in respiratory failure, cardiac injury, acute respiratory distress syndrome and even death [7].

Since September 1^st^, 2020, there have been 25,298,875 confirmed cases and 847,602 deaths (29.8% fatality rate) of COVID-19 across the globe [16]. Locally, 54,008 confirmed cases and 1,013 (1.09% fatality rate) have been reported in Nigeria by the Nigeria Centre for Disease Control (NCDC) [17]. The incident case of COVID-19 in Nigeria was recorded on February 27^th^, 2020 in a 44-year-old Italian citizen that came to Nigeria via Murtala Muhammed Airport on February 24^th^, 2020 from Milan, Italy [18]. The assessment of knowledge, perception, preventive practice and effect are important for public health response to epidemics/pandemics as it helps to show the willingness of the population to accept behavioral changes that proceeds with the epidemic/pandemic as well as direct areas of further effort in combating the same and future epidemics. Hence, the purpose of this study is to assess knowledge, perception, preventive practice on COVID-19 and as well evaluate proportion of the economic impact on them.

## Methods

**Study design:** this survey study used a descriptive cross-sectional approach. The knowledge, perception, practice of Nigerians towards COVID-19 and the possible economic effect on them were assessed via a questionnaire.

**Study site:** data collection was performed online. The study started from May 25^th^, 2020 to June 25^th^, 2020.

**Sampling and sample size:** snowball sampling technique was used to invite study participants. Convenient sampling was used.

**Recruitment procedure:** the call for participation was made on social media. Facebook and WhatsApp were used as they are the most popular social media platforms in Nigeria [19]. Only Nigerians currently residing in Nigeria were eligible to participate.

**Data collection:** the survey was conducted online using Google form (Annex 1). A self-made questionnaire was used to collect demographic and epidemiological information of participants including their knowledge, perception, preventive practice and the impact of COVID-19 on them. The questionnaire consisted of five main categories: demographic, knowledge, perception, preventive practice and impact of COVID-19. Socio-demographic variables included age, gender, religious affiliation, denomination for Christians, state of origin, monthly income range, occupation, marital status and level of education.

**Variable measurement:** the knowledge about COVID-19 was adapted according to previous researches [7]. To evaluate the knowledge of the participants, 13 items were listed as questions pertaining to the knowledge of pathogen, mode of transmission and treatment options and control. Participation was given “true” and “false” and other cases “yes” and “no” options. Correct answers were scored 1 point while wrong answers/not sure answers were scored 0. Perception was reported as frequencies and percentages and was not scored as scientific validity might be pertinent to rule them null. Practice and impact were represented in frequencies and percentages. Overall knowledge status was ranked using 3 labels: low (<7/14), moderate/average (7-9.9) and high (≥10) following the quartile ranking of the 13 questions. A quartile score of 10 or more correct answers were used as cut off point for high knowledge, while scores less than 7 were stratified as low knowledge.

**Validity and reliability:** the validity and reliability of the questionnaire items were carried out via pilot study that was conducted on 20 participants. The Cronbach´s alpha test result was 0.785 for the knowledge analysis.

**Statistical analysis:** data generated in this study were exported to Excel sheet 2010 and later exported to SPSS version 20 for statistical analysis. Descriptive demographics were represented in frequencies and percentages. Descriptive statistics, t-test, one-way ANOVA and bivariate logistics regression were used to analyze data. Alpha value was set at 0.05.

**Data availability:** data used for this study is available from the corresponding author on request.

**Ethical approval:** ethical approval for this study was obtained from Cross River State, Ministry of Health. Anonymity and confidentially were strictly maintained. Respondents were made to grant consent by clicking the approval question before proceeding to answer the questionnaire.

## Results

A total of 508 participants (Nigerians currently residing in Nigeria) filled the form. Among the participant, 279 (54.9%) were males while 229 (45.1) were females. The age groups <18 years, 18-28 years, 29-38 years, 39-48 years, 49-58 years and >59 years accounted for 1.4% (n=4), 31.9% (n=162), 37.8% (192), 21.3% (n=108), 5.3% (n=27) and 2.4% (n=12) of participants respectively. While polytechnics/college of education/undergraduates (n=258; 50.8%) and post graduates (n=227; 44.7%) represented the majority of participants, those who attended only primary school (n=1; 0.2%) and those who attended no formal education/artisans (n=3; 0.6%) were the least numerous. Secondary school graduates accounted for 3.7% (n=19) of participants. Singles, married, divorced, widowed, separated couples and cohabiting persons accounted for 48.4% (n=246), 48.8% (n=248), 0.2 (n=1), 2.0% (n=10), 0.2% (n=1) and 0.4% (n=2) of participants, respectively. The majority of participants (94.2%; n=475) were Christians, Muslims, African traditional religion adherents, atheist, agonists, others made up 3.2% (n=16), 0.2% (n=1), 1.6 (n=8), 0.4% (n=2) and 0.4% (n=2) of participants, respectively. Among the Christians, Pentecostals (36.3%; n=169), and Catholics (36.1%; n=168) represented the majority of participants. Other Protestants/Orthodox churches, Anglicans and others accounted for 24.5% (n=114), 2.6% (n=12) and 0.6% (n=3) of participants, respectively. The larger proportion of participants were civil/public servants (42.9%; n=218), students (24.0%, n=122) and private business owners and workers (22.8%; n=116). On the other hand, unemployed persons, farmers, retirees and other professions made up 6.7% (n=34), 1.4% (n=7), 0.2% (n=1) and 1.8% (n=9) of participants, respectively. Participants who earned less than 100,000 naira monthly income accounted for 52.1% (n=228) of participants while those who earned between 100,000 to 200,000 accounted for 26.5% (n=116) of participants. Those who earned monthly income of more than 200,000 made up 21.5% (n=94) of participants (Table 1). The evaluation of knowledge score of respondents regarding their knowledge of COVID-19 showed 98.03% had high knowledge of COVID-19 while 1.97% had moderate knowledge.

**Table 1:** demographic characteristics of the participants

Demographic characteristics		Frequency (%)
Age group (years)	<18	7 (1.4)
	18-28	162 (31.9)
	29-38	192 (37.8)
	39-48	108 (21.3)
	49-58	27 (5.3)
	≥59	12 (2.3)
Gender	Male	279 (54.9)
	Female	229 (45.1)
Level of education	No formal education/artisan	3 (0.6)
	Primary education	1 (0.2)
	Secondary education	19 (3.7)
	Polytechnic/college of edu./Undergrad	258 (50.8)
	Postgraduate education	227 (44.7)
Marital status	Single	246 (48.4)
	Married	248 (48.8)
	Divorced	1 (0.2)
	Widowed	10 (2.0)
	Separated	1 (0.2)
	Cohabiting	2 (0.4)
Occupation	Civil/public servants	218 (42.9)
	Students	122 (24.0)
	Business/private sector	116 (22.8)
	Unemployed	34 (6.7)
	Farmers	7 (1.4)
	Retirees	1 (0.2)
	Others	9 (1.8)
Monthly income range (Naira)	<100,000	228 (52.1)
	100,000 - 200,000	116 (26.5)
	≥200,000	94 (21.5)
	Not disclosed	70 (13.8)
Religion	Christianity	475 (94.2)
	Islam	16 (3.2)
	African traditional religion	1 (0.2)
	Atheism	8 (1.6)
	Agnostics	2 (0.2)
	Others	6 (0.4)
Christian denomination	Catholics	168 (36.1)
	Anglicans	12 (2.6)
	Other protestants/Orthodox	114 (24.5)
	Pentecostals	169 (36.3)
	Others	3 (0.6)
Region	North	43 (8.5)
	South	172 (33.9)
	West	29 (5.7)
	East	264 (52.0)

Table 2 shows the knowledge of participants about COVID-19. Approximately 99.8% of participants had heard about COVID-19 while 0.2% (n=1) hadn´t heard about it. While 93.7% (n=476) of respondents were aware that COVID-19 is a viral infection, 1.0%, (n=5), 1.0% (n=5), 0.2% (n=1) and 0.6 (n=3) believed that 5G network, bacteria, fungi and God´s punishment were the cause of COVID-19 pandemic. While 99.8% (n=50.7) of participants selected respiratory droplets as means of infection, 14.4% (n=73) believed it was sexually transmitted. Mosquito bites and water were also selected by 0.2% (n=1) and 6.7% (n=34%) of respondents. Approximately 91.9% (n=467) believed that COVID-19 does not affect blacks while 8.1% (n=41) selected otherwise.

**Table 2:** summary of participants’ knowledge of COVID-19

Question	Response	Frequency (%)
Have you heard of COVID-19	Yes	507 (99.8)
	No	1 (0.2)
COVID-19 does not affect blacks	Yes	467 (91.9)
	No	41 (8.1)
People of any age can be infected with COVID-19	Yes	501 (98.6)
	No	7 (1.4)
Severe symptoms are likely to develop in the elderly and those with underlying disease	Yes	498 (98.0)
	No	10 (2.0)
Incubation period varies between 2-14 days	Yes	462 (90.9)
	No	46 (9.1)
COVID-19 is curable	Yes	317 (62.4)
	No	191 (37.6)
COVID-19 can be prevented by regular washing of hands	Yes	505 (99.4)
	No	3 (0.6)
COVID-19 can be prevented by social distancing	Yes	507 (99.8)
	No	1 (0.2)
COVID-19 can be prevented by avoiding contact with sick people	Yes	463 (91.1)
	No	45 (8.9)
COVID-19 cannot be prevented by wearing face mask	Yes	115 (22.6)
	No	393 (61.0)
What is the cause of COVID-19	5G network	5 (1.0)
	Viral infection	476 (93.7)
	Bacterial infection	5 (1.0)
	Fungal infection	1 (0.2)
	God's punishment	3 (0.6)
	I don't know	18 (3.5)
How does COVID-19 mostly spread	Sexual route	73 (14.4)
	Air droplets	507 (99.8)
	Mosquito bite	1 (0.2)
	Contaminated water	34 (6.7)
Thick the symptoms of COVID-19	Correct answer	497 (97.8)
	Wrong answer	11 (2.2)

Note: those who ticked at least 4 principal symptoms (fever, fatigue, dry cough and dyspnea) out of the 13 listed options (sneezing, stooling, fever, vomiting, catarrh and running nose, excessive sweating, headache, loss of smell, dry cough, body weakness, abdominal pain, bleeding, difficulty in breathing) were considered correct

Table 3 shows the summary of respondents´ preventive practices towards COVID-19. The majority of (95.1%) respondents made use of nose masks. Regarding stratification of the type of nose mask used, 57.8%, 33.5%, 0.2% and 0.2% of respondents that used mask used locally made fabric masks, surgical masks, N95 masks and 3M masks respectively, while 1.0%, 0.2% and 0.2% combined both locally made fabric masks with surgical masks, locally made fabric masks with N95 and combination of the four mask types. Regarding the use of alcohol-based hand sanitizer, the majority (78.9%) of respondents had and used alcohol-based hand sanitizers. Regarding further probes, 85.7% and 14.3% of respondents used pharmaceutically made hand sanitizers and locally made (improvised) hand sanitizers, respectively. Approximately 37.6% of respondents reported defying the lockdown ban on social gathering to attend religious worship. The majority of the respondents washed their hands each time they returned from outside (48.0%), followed by those who washed their hands more than five time daily (42.3%). Those who washed their hands 2 times, 3 times, 4 times and 5 times daily accounted for 1.4%, 2.6%, 3.1% and 1.2% of respondents, respectively.

**Table 3:** summary of participants’ preventive practices against COVID-19

Question	Response	Frequency (%)
Do you currently use face mask	Yes	483 (95.1)
	No	25 (4.9)
If your answer is “yes” which type	Local fabric made	279 (57.8)
	Surgical mask	162 (33.5)
	N95 mask	1 (0.2)
	3M mask	1 (0.2)
	Fabric + surgical	5 (1.0)
	Fabric + N95	1 (0.2)
	All of the above	1 (0.2)
Do you currently carry and use alcohol-based hand sanitizer	Yes	401 (78.9)
	No	107 (21.1)
If your answer to the above is “yes”, what is the source	Pharmaceutically made	349 (85.7)
	Locally made (improvised)	58 (14.3)
Have you attended religious worship since the lock down ban on social gathering	Yes	191 (37.6)
	No	317 (62.4)
How often do you wash your hand in a day?	2 times a day	7 (1.4)
	3 times a day	13 (2.6)
	4 times a day	16 (3.1)
	5 times a day	6 (1.2)
	More than 5 times a day	215 (42.3)
	Each time you return from outside	244 (48.0)

Table 4 shows the analysis of knowledge score about COVID-19 in view of the demographic characteristics of the respondents. The variation in knowledge score was assessed using ANOVA and t-test. The knowledge score was only found to differ significantly across marital status. The knowledge score of single and married respondents was significantly higher than of the rest of the respondents. The knowledge score across age, gender, level of education, occupation, monthly income, religion, religious denomination and region were comparable.

**Table 4:** analysis of knowledge score by demographic characteristics

Demographic characteristics		Frequency	Knowledge score (SD)	F/t value	P-value
Age group (years)	<18	7	11.4 (1.3)	1.971	0.079
	18 - 28	162	11.9 (0.9)		
	29 - 38	192	11.7 (1.0)		
	39 - 48	108	11.5 (1.0)		
	49 - 58	27	11.6 (1.0)		
	≥59	12	11.6 (1.1)		
Gender	Male	279	11.8 (0.9)	0.588	0.444
	Female	229	11.7 (1.0)		
Level of education	No formal education/artisan	3	11.3 (0.6)	0.990	0.412
	Primary education	1	10.0 (0.0)		
	Secondary education	19	11.8 (0.7)		
	Polytechnic/college. of education/undergrad	258	11.7 (1.0)		
	Postgraduate education	227	11.7 (0.9)		
Marital status	Single	246	11.9 (1.0)	4.067	0.001
	Married	248	11.6 (1.0)		
	Divorced	1	12.0 (0.0)		
	Widowed	10	11.1 (0.7)		
	Separated	1	13 (0.0)		
	Cohabiting	2	11.5 (0.7)		
Occupation	Civil/public servants	218	11.7 (1.0)	0.815	0.558
	Students	122	11.9 (0.9)		
	Business/private sector	116	11.6 (0.9)		
	Unemployed	34	11.7 (1.1)		
	Farmers	7	11.4 (0.5)		
	Retirees	1	12 (0.0)		
	Others	9	11.7 (0.8)		
Monthly income range (Naira)	<100,000	228	11.7 (1.0)	.100	0.960
	100,000 - 200,000	116	11.7 (0.7)		
	≥200,000	94	11.7 (1.0)		
	Not disclosed	70	11.8 (1.0)		
Religion	Christianity	475	11.7 (1.0)	0.689	0.632
	Islam	16	11.9 (0.9)		
	African traditional religion	1	12.0 (0.0)		
	Atheism	8	11.7 (0.5)		
	Agnostics	2	12.5 (0.7)		
	Others	6	12.5 (0.7)		
Christian denomination	Catholics	168	11.8 (0.9)	1.525	0.194
	Anglicans	12	11.7 (0.0)		
	Other protestants/Orthodox	114	11.7 (1.0)		
	Pentecostals	169	11.6 (1.0)		
	Others	3	11.3 (0.9)		
Region	North	43	11.8 (0.9)	0.934	0.970
	South	172	11.7 (1.0)		
	West	29	12.0 (0.9)		
	East	264	11.7 (1.0)		

Table 5 shows the summary of participants´ perception of COVID-19. A proportion (6.5%) of respondents believed that COVID-19 could be prevented by eating bitter cola. Approximately 4.7% of the respondents believed that drinking alcohol (especially the local gin) could prevent contraction of COVID-19. Eating of ginger and garlic was perceived by 26.8% of the respondents as a means of preventing COVID-19 infection. Almost a quarter (30.1%) of the respondents believed that COVID-19 could be prevented by regular prayers and spiritual protection. Bathing of hot water was perceived by 13.4% of the respondents as a means of preventing COVID-19. Approximately 22.8% of respondents had the perception that COVID-19 couldn´t be prevented except if God help. The majority of the respondents (61.0%) doubted on the number of cases and deaths attributed to COVID-19 by the Nigeria Centre for Disease Control. In similar vein, the majority (63.0%) of respondents believed that COVID-19 pandemic in Nigeria had been politicized. Only a small proportion (35.8%) of respondents believed they were at risk of contracting COVID-19. Approximately 48.4% of respondents supported the glamour to reopen worship centers. A high proportion (44.5%) of respondents believed COVID-19 originated from Chinese laboratory. Approximately half (50.2%) of respondents said that they had confidence in Nigerian Center for Disease Control strategy against COVID-19 while the remaining ones doubted about the efforts of the agency to fight against the pandemic. Approximately 7.3% of respondents believed that COVID-19 was not in Nigeria, and that the announcement was a hoax.

**Table 5:** summary of participants’ perception of COVID-19

Question	Response	Frequency (%)
COVID-19 can be prevented by eating bitter cola	Yes	33 (6.5)
	No	475 (93.5%)
COVID -19 can be prevented by drinking alcohol (ogogoro)	Yes	24 (4.7)
	No	484 (95.3)
COVID-19 can be prevented by eating ginger and garlic	Yes	136 (26.8)
	No	372 (73.2)
COVID-19 can be prevented by regular prayers and spiritual protection	Yes	153 (30.1)
	No	355 (69.9)
COVID-19 can be prevented by bathing hot water	Yes	68 (13.4)
	No	440 (86.6)
COVID-19 cannot be prevented, only God can help	Yes	116 (22.8)
	No	392 (77.2)
Do you believe in the number of sick and dead persons owing to COVID-19 reported by NCDC	Yes	198 (39.0)
	No	310 (61.0)
In your opinion do you think COVID-19 has been politicized	Yes	320 (63.0)
	No	46 (9.1)
	I don't know	142 (28.0)
Do you think you are at risk of COVID-19 infection	Yes	182 (35.8)
	No	258 (50.8)
	God won't allow it	68 (13.4)
Do you support the clamour to reopen worship places	Yes	246 (48.4)
	No	262 (51.6)
What is the origin of COVID-19	Chinese laboratory	226 (44.5)
	American military	5 (1.0)
	Effect of 5G network	4 (0.8)
	Animal to human	179 (35.2)
	Divine punishment	3 (0.6)
	I don't know	91 (17.9)
Do you have confidence on NCDC and ministry of health in the fight against COVID-19	Yes	255 (50.2)
	No	253 (49.8)
COVID-19 is not in Nigeria, it is a hoax	True	36 (7.3)
	False	459 (92.7)

Table 6 shows socio-demographic predictors of major preventive behaviors. Gender, level of education, monthly income, Christian denomination and region of the respondents were the major predictors of use of hand sanitizer. Age, level of education, monthly income, Christian denomination and region of the respondents predicted worship center attendance during the lockdown ban on religious gathering. None of socio-demographic variables predicted face mask use. With regard to predictors of alcohol-based hand sanitizer use, males and those who attended secondary school used averagely 1.7 and 7.3 times less frequently hand sanitizer compared to their counterparts (females and postgraduate fellows). Respondents who earned >N200,000 monthly income were averagely 3.6 times more likely to use hand sanitizer than their counterparts who didn´t disclose their income. Christian respondents who were Catholics, other Protestants/Orthodox and Pentecostals were approximately 7.6, 15.0 and 6.2 times more likely to use hand sanitizer than their other Christian denomination counterparts, respectively. Respondents from the south and west region of the country were averagely 1.8 and 1.3 times less likely to use hand sanitizer compared to their counterparts from the east, respectively.

**Table 6:** socio-demographic predictors of major preventive behaviors

Demographic characteristics	Use of nose mask		Use of hand sanitizer		Attendance of worship center	
Age group (Years)	OR (95%CI)	P-value	OR (95%CI)	P-value	OR (95%CI)	P-value
<18	0 (0)	0.999	2.000 (0.166 - 24.069)	0.585	3.000 (0.263 - 34.198)	0.376
18 - 28	0 (0)	0.999	0.747 (0.194 - 2.876)	0.671	0.362 (0.105 - 1.250)	0.108
29 - 38	0 (0)	0.999	1.308 (0.338 - 5.060)	0.698	0.191 (0.055 - 0.660)	0.009
39 - 48	0 (0)	0.999	2.939 (0.691 - 12.504)	0.144	0.318 (0.090 - 1.123)	0.075
49 - 58	0 (0)	0.999	2.667 (0.452 - 15.722)	0.279	0.538 (0.130 - 2.223)	0.392
≥59	1		1		1	
**Gender**						
Male	1.708 (0.768 - 3.795)	0.189	0.575 (1.115 - 2.709)	0.015	0.847 (0.591 - 1.215)	0.367
Female	1		1		1	
**Level of education**						
No formal education/artisan	5.163E7 (0)	0.999	3.795E8 (0)	0.999	0.829 (0.074 - 9.286)	0.879
Primary education	5.163E7 (0)	1.000	3.795E8 (0)	1.000	2.679E9 (0)	1.000
Secondary education	0.575 (0.067 - 4.938)	0.614	0.137 (0.051 - 0.369)	<0.01	3.594 (1.317 - 9.810)	0.013
Poly/coll of edu./undergrad.	0.428 (0.175 - 1.044)	0.062	0.935 (0.596 - 1.467)	0.771	0.899 (0.620 - 1.302)	0.572
Postgraduate education	1		1		1	
**Marital status**						
Single	0 (0)	0.999	0 (0)	0.999	0.577 (0.036 - 9.336)	0.699
Married	0 (0)	0.999	0 (0)	0.999	0.600 (0.037 - 9.707)	0.719
Divorced	1 (0)	1.000	1 (0)	1.000	0 (0)	1.000
Widowed	1 (0)	1.000	0 (0)	0.999	2.333 (0.107 - 50.982)	0.590
Separated	1 (0)	1.000	1 (0)	1.000	0 (0)	1.000
Cohabiting	1		1		1	
**Occupation**						
Civil/public servants	6.371 (0.655 - 61.983)	0.111	2.608 (0.461 - 14.762)	0.278	1.108 (0.199 - 6.180)	0.907
Students	2.280 (0.240 - 21.425)	0.471	1.379 (0.241 - 7.883)	0.718	2.203 (0.389 - 12.473)	0.372
Business/private sector	4.040 (0.394 - 41.409)	0.240	2.289 (0.391 - 13.420)	0.359	0.753 (0.131 - 4.336)	0.751
Unemployed	2.067 (0.178 - 24.006)	0.562	0.633 (0.102 - 3.938)	0.624	0.615 (0.095 - 4.006)	0.611
Farmers	3.230E (0)	0.999	3.000 (0.199 - 45.244)	0.437	5.000 (0.472 - 52.961)	0.181
Retirees	3.230E (0)	1.000	8.077E8 (0)	1.000	0 (0)	1.000
Others	1		1		1	
**Monthly income range (Naira)**						
<100,000	0.707 (0.231 - 2.163)	0.543	1.526 (0.838 - 2.779)	0.167	0.501 (0.291 - 0.860)	0.012
100,000 - 200,000	3.455 (0.616 - 19.374)	0.159	1.484 (0.758 - 2.905)	0.250	0.427 (0.232 - 0.783)	0.006
≥200,000	2.788 (0.496 - 15.672)	0.244	3.600 (1.568 - 8.267)	0.003	0.376 (0.198 - 0.714)	0.003
Not disclosed	1		1		1	
**Religion**						
Christianity	0 (0)	0.999	0 (0)	0.999	0.630 (0.039 - 10.136)	0.745
Islam	1.0 (0)	1.000	0 (0)	0.999	0.333 (0.017 - 6.654)	0.472
African traditional religion	1.0 (0)	1.000	1 (0)	1.000	0 (0)	1.000
Atheism	0 (0)	0.999	0 (0)	0.999	0.143 (0.004 - 4.612)	0.272
Agnostics	1.0 (0)	1.000	1 (0)	1.000	0 (0)	0.999
Others	1		1		1	
**Christian denomination**						
Catholics	0.649 (0.080 - 5.272)	0.939	7.670 (3.00 - 19.611)	<0.01	0.272 (0.111-0.670)	0.005
Anglicans	7.343E7 (0)	0.686	3.129E9 (0)	0.999	0.214 (0.045-1.012)	0.052
Other protestants/Orthodox	1.080 (0.115 - 10.139)	0.999	15.00 (5.183 - 43.409)	<0.01	0.630 (0.250-1.589)	0.328
Pentecostals	0.783 (0.095 - 6.481)	0.947	6.217 (2.449 - 15.782)	<0.01	0.401 (0.164-0.981)	0.045
Others	1		1		1	
**Region**						
North	1.148 (0.252 - 5.238)	0.960	0.776 (0.348 - 1.731)	0.536	1.220 (0.633-2.349)	0.553
South	1.148 (0.471 - 2.798)		0.546 (0.344 - 0.869)	0.011	1.191 (0.804-1.764)	0.384
West	0.756 (0.163 - 3.505)		0.788 (0.303 - 2.045)	0.011	0.271 (0.092-0.802)	0.018
East	1		1		1	
**Knowledge classification**						
Average knowledge	8.898E7 (0)	0.999	0.616 (0.157 - 2.423)	0.488	0.707 (0.181-2.766)	0.618
High knowledge	1		1		1	

With regard to predictors of defying lockdown order to attend worship places, respondents who were within 29-38 years and those from western region were approximately 5.2 and 3.7 times less likely to attend worship centers during lockdown ban than their counterparts (subjects aged >59 years) and subjects from the eastern region, respectively. With regard to predictor of education level, respondents who attended secondary school were approximately 3.6 times more likely to attend worship center during lockdown ban when compared to their postgraduate counterparts. Those who earned <N100,000, N100,000-N200,000 and >N200,000 monthly income were approximately 2.0, 2.3 and 2.7 times more likely to avoid attendance at worship centers during lockdown ban when compared to their counterparts who didn´t disclose their income. With regard to Christian denominations, Catholics, other Orthodox/protestants and Pentecostals were 200.0, 3.0 and 22.2 times less likely to attend worship centers during lockdown ban on social gathering when compared to others.

**Figure 1:**
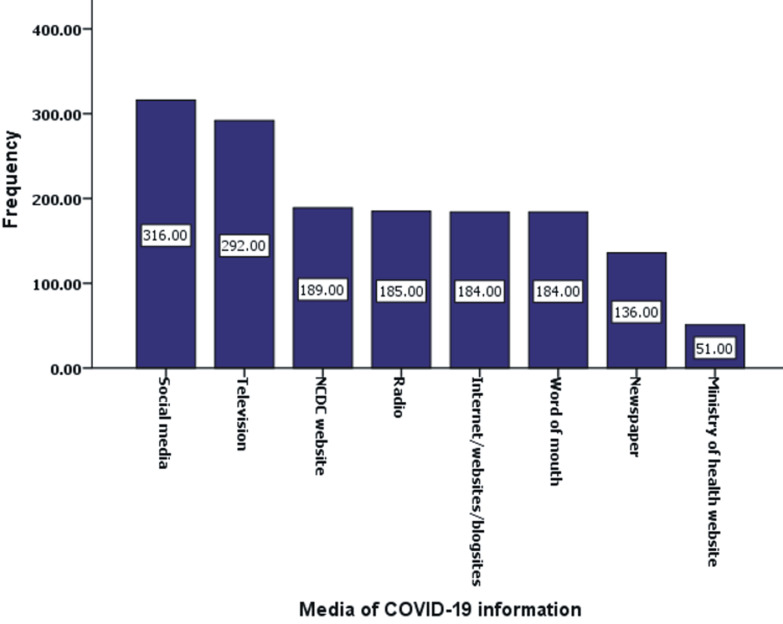
means through which respondents got information about COVID-19

Figure 1 shows the media through which the respondents heard about COVID-19. Approximately 62.2% admitted hearing about COVID-19 via social media followed by 57.5% that had gotten information about the pandemic via television. Other means through which the respondents heard about the pandemic were: NCDC website (37.2%), radio (36.4%), internet/websites/blog sites (36.2%), word of mouth from colleagues, family and friends (36.2%), newspapers (26.7%) and ministry of health website (10.0%). The analysis of the effects of the pandemic on respondents´ job showed that approximately 69.5% (n=353) of respondents were in jobs affected by the pandemic. Further analysis showed that approximately 66.7%, 20.9% and 12.4% of affected person had experienced job loss, pay/salary/allowance and patronage cut, respectively.

## Discussion

Coronavirus disease 2019 (COVID-19), though a novel virus, has laid a strong epidemiological landmark within the short period since it was reported in December 2019. Considering that the virus is novel, with unknown epidemiological dimension, it becomes pertinent for health authorities and policy makers to have idea of people´s knowledge, perception, preventive practices and effect of the pandemic on the citizens.

The average knowledge score of Nigerian respondents in regards to COVID-19 was 11.7 ± 1.0 (9-13), which corresponded to high knowledge. The majority of respondents (98.0%) had high knowledge about COVID-19. This finding is a reflection of COVID-19 information landscape in Nigeria. The Nigerian government through the Nigerian Center for Disease Control after confirmation of the index case on February 27^th^, 2020, embarked on several interventions geared towards public enlightenment campaign which included daily update on COVID-19 information and development of jingles in 7 local languages [18]. More so, prior and concomitant epidemics, such as Monkeypox and Lassa fever [20,21], that preceded COVID-19 in Nigeria, might have shaped the immediate and willingness to imbibe information about the pandemic. Knowledge score was comparable among respondents in the various demographic categories, except for marital status, where the knowledge score was statistically significant. This is possibly due to skewed frequency distribution for single and married respondents, with only few scores representing the remainder of the marital status.

We also evaluated the frequency of individuals who adhered to certain beliefs that are in fashion in the wake of COVID-19 pandemic. Just like previous pandemics, there have been varying “infodemics” concerning COVID-19 which occurred in the form of rumors and conspiracy theories concerning the origin of the pandemic and treatment options [22-24]. Rumors heard by the respondents included the assertion that COVID-19 could be prevented by eating bitter cola, ginger, garlic, alcohol, bathing hot water and even prayers. All these claims have not been substantiated (although the converse hasn´t been substantiated in a randomized clinical trial). There was also the conspiracy theory suggesting COVID-19 originated from Chinese laboratory and the sub-population that believe COVID-19 originated from 5G network. These unverified perceptions are not peculiar to respondents´ location (Nigeria), but the same has been reported across the globe [22]. Previous studies have documented conspiracy theories about Zika virus being a biological weapon during the 2015-2016 outbreak [25]. For instance, the believe about alcohol as a preventive and curative measure against COVID-19 has been reported to have led to the death of 800 persons, hospitalization of 5,876 and development of complete blindness in 60 persons after drinking methanol in Iran [26-29]; 30 deaths in Turkey [30]; and 2 in Qatar [31]. A proportion of the respondents believed that COVID-19 was a hoax and not in Nigeria. This type of perception was also documented during the Ebola epidemic [31,32] as well as the onset of HIV epidemic [33].

Nearly half of the respondents declined not having confidence in the government´s efforts in fighting COVID-19. More than half believed that the pandemic report had been politicized while a reasonable proportion doubted about the scores of morbidity and mortality reported by the government agency (NCDC) accredited to COVID-19. Trust on the government by citizen is paramount in controlling epidemics and this reflects a more task ahead for government agencies to gain the trust of the populace to make actualization of her efforts a reality. Having a strong faith in the government agency (NCDC) will improve public health masses compliance.

The preventive practices recorded by the respondents included the use of face mask, of alcohol-based hand sanitizer and the avoidance of worship places (as advised by the government). Gender, level of education, monthly income, and Christian denomination were predictors of use of alcohol-based hand sanitizer. Females were more likely to use hand sanitizer than their male counterparts. This finding is corroborated by previous studies that attributed higher hand hygiene to females than males [34,35]. A qualitative research is needed to explicitly find the cause of this gender disparity in hand hygiene. However, To *et al*. [36] reported that factors such as business, tiredness discouraged males from practicing better hand hygiene than their female counterparts while Suen *et al*. attributed the same to males being in haste, hence, forgetting/ignoring hand hygiene while in a hurry [34]. In this study respondents with postgraduate level of education were more likely to carry and use hand sanitizer. In a similar manner, a study in Vietnam had reported association of higher education with good hand hygiene [36]. We too observed higher odd of use of hand sanitizer in those with higher income classes. This finding are in line with that from previous study on hand hygiene [36]. This might be linked to affordability to hand sanitizer. Approximately 85.7% of those who used hand sanitizer used pharmaceutical sanitizer while 14.3% had to improvise. The cost of pharmaceuticals such as hand sanitizer increased as much as 300% [37]. On the other hand, level of education, monthly income, Christian denomination and region were predictors to attendance to worship centers during lockdown. Lower level of education was associated with attendance to worship centers during ban on worship centers (during lockdown). Nigerians are highly religious people and often put their religion above every other thing [38]. Only a higher level of education could possibly lead most people to weigh breaking the law rather than taking on religious obligations. The Pentecostals were more likely to break down lock down rules to attend religious worship than the traditional and Orthodox churches. Pentecostal churches in Nigeria are mostly individually owned and the law that govern them is mostly based on the founder´s (General Overseer) view. Respondents from the western region of Nigeria were less likely to break lockdown rules to attend religious centers. An observatory study will be essential to unravel the actual reason for this trend. Respondents within the age bracket of 29-38 years were less likely to break down lock down rule to attend worship centers than their aged counterparts in ≥59 years´ category. Similar trends were reported by a study in Malaysia [39]. They attributed this trend to observation of cultural norms in aged persons despite the health risks.

Social media contributed to the majority of the route of awareness of COVID-19. This observation serves as a guide to policy makers in disseminating information to public health currently and in future. COVID-19 pandemic affected job of approximately 69.5% of respondents in different ways: job loss, pay/salary/allowance and low patronage cut. The devastating effect of COVID-19 globally, especially on employment status, has been documented. It hardly spared any country [40].

However, this study is subject to some potential limitations. First, our participants needed access to internet via smart phone or computer to fill the form, hence, this may have potentially led to selection bias. However, our sample was fairly representative of general Nigeria population. Secondly, there is the possibility that the same respondents gave answers based on what they perceived was expected of them in terms of practice since they were self-reported; though we made maximum effort to make responses anonymous.

## Conclusion

The study findings suggest that Nigerians have an optimum knowledge of COVID-19, although the majority of them does not have faith in the efforts of NCDC and ministry of health in combating the pandemic. A good number of the respondents engaged in preventive practices. Gender, level of education, monthly income, and Christian denomination were found to be associated with use of hand sanitizer while age, level of education, monthly income. Christian denomination and religion were predictors for attendance of worship centers during lockdown. This information would be useful to policy makers in making emphasis while channeling public health information.

### What is known about this topic

- Coronavirus disease 2019 has been declared pandemic by the World Health Organization;
- The COVID-19 pandemic has altered socio-economic aspects of human life globally;
- The pattern of knowledge, attitude, perception and health practices of a population affects the impact of epidemics.

### What this study adds

- Knowledge of COVID-19 among Nigerians studied is high: knowledge score was comparable among all groups (age, gender, ethnicity, monthly income, level of education, religious inclination) except marital status;
- Predictors of preventive practices among the studied population includes: gender, level of education, monthly income and Christian denomination;
- COVID-19 has led to loss of jobs/reduced patronage in most of the studied Nigerians.

## Annex

**Annex 1**: sample of study questionnaire (PDF-229Kb)
